# Characterization of Mineral Composition and Nutritional Value of *Acacia* Green Pods

**DOI:** 10.3390/plants12091853

**Published:** 2023-04-30

**Authors:** Soraia I. Pedro, Carlos A. L. Antunes, Carmo Horta, Inês Pitacas, Joana Gonçalves, Jorge Gominho, Eugenia Gallardo, Ofélia Anjos

**Affiliations:** 1 Centro de Biotecnologia de Plantas da Beira Interior (CBPBI), 6000-098 Castelo Branco, Portugal; soraia_p1@hotmail.com; 2 Centro de Estudos Florestais (CEF), Laboratório Associado TERRA, Instituto Superior de Agronomia, Universidade de Lisboa, 1349-017 Lisboa, Portugal; jgominho@isa.ulisboa.pt; 3 Instituto Politécnico de Castelo Branco (IPCB), 6000-084 Castelo Branco, Portugal; carlosalbertoantunescb@gmail.com; 4 CERNAS-IPCB Research Centre for Natural Resources, Environment and Society, Instituto Politécnico de Castelo Branco, 6000-084 Castelo Branco, Portugal; carmoh@ipcb.pt (C.H.); inespitacas@ipcb.pt (I.P.); 5 Centro de Investigação em Ciências da Saúde (CICS-UBI), Universidade da Beira Interior, 6200-506 Covilhã, Portugal; joanadgoncalves13@gmail.com; 6 Laboratório de Fármaco-Toxicologia—UBIMedical, Universidade da Beira Interior, 6200-506 Covilhã, Portugal

**Keywords:** *Acacia* species, green pods, nutritional value, elemental analysis, NIR

## Abstract

The *Acacia* genus is considered one of the most invasive taxa in some habitats, namely coastal dunes, maritime calcareous soils, fresh lands in the valleys, mountainous areas, and the banks of watercourses and roadsides. In Portugal, the severity risk is very high, so this study aimed to evaluate the nutritional and mineral contents of the green pods as a potential source for livestock feeds and soil fertilizer because, as far as we know, there is no use for this species. The seven different species of *Acacia* (*Acacia mearnsii* Link, *Acacia longifolia* (Andrews) Willd, *Acacia melanoxylon* R. Br., *Acacia pycnantha* Bentham, *Acacia dealbata* Link., *Acacia retinodes* Schlecht, and *Acacia cyclops* A. Cunn. ex G. Don fil) were evaluated. The results showed that *Acacia* green pods have a high protein, fibre and minerals content, especially in potassium (K), calcium (Ca) and magnesium (Mg). All species present a different profile of the studied parameters, suggesting different potentials for their future use. Near-infrared spectroscopy was a potential tool to predict the earlier quality of the *Acacia* green pods to better select the raw material for the different applications.

## 1. Introduction

*Acacia* sp. is an aggressive invasive species which is widespread throughout the world, representing a threat to biodiversity and productivity of the forestry sector [1,2]. These plants are distributed throughout the Portuguese territory. Concerning other countries, they are widespread in a wide variety of environments, including arid zones, coasts, sub-alpine zones, native forests, and agricultural lands, modifying the structure of ecosystems [2,3]. These invasive species can spread rapidly and affect the plant composition of communities, reduce the regeneration rates of native species, and stress landscape structures, among other implications [4]. Measures for its eradication or control are expensive and have been considered unfeasible from practical and economic perspectives. For this reason, studies have been performed on the sustainable valorization of the waste of these species [1,5,6,7].

Studies report that the individual parts of *Acacia* sp., such as bark, wood, leaves, flowers, pods, seeds, or roots, are rich sources of bioactive secondary metabolites (e.g., amines and alkaloids, cyanogenic glycosides, cyclitols, fats, seed acids and oils, gums, non-protein amino acids, terpenes, tannins and other flavonoids, and simple phenols) [7,8,9] and have been used in traditional medicine to treat a wide range of diseases, such as diabetes and cancer. In addition, other important biological activities, such as anti-inflammatory, antiviral, antidiabetic, immunomodulatory, hepatoprotective, and anthelmintic, have already been reported for extracts obtained from *Acacia* sp. [10,11,12,13,14].

Studies on the bioactive properties of pods from invasive *Acacia* sp. in Portugal are scarce, and to our knowledge, the focus has been mainly on their allelopathic effects. The authors [15,16] observed that extracts of *A. dealbata*, *A. cyclops*, *A. mollissima*, and *A. saligna* pods had demonstrated the allelopathic ability of a water extract against lettuce (*L. sativa*). Puga et al. [17] focused their study on the potential antioxidant properties of extracts of *A. shaffneri* and *A. farnesiana*, and observed protection against oxidation-induced damage in pig kidney cells LLC-PK1. Other authors have reported that pod extracts presented good antimicrobial activity [18,19], antidiabetic and hypolipidemic effects [20], antihyperglycemic properties [21], antihypertensive and antispasmodic actions [22], and improved wound healing by relieving oxidative stress and suppressing pro-inflammatory cytokines [23].

On the other hand, *A. farnesiana* is easily consumed by livestock [24,25], which has led to the publication of studies on the use of these trees as a forage resource. 26. Barrientos-Ramírez et al. [26] and Walker [27] found that the pods of *Leucaena diversifolia* and *L. leucocephala* have sufficient protein and dry matter content to meet the needs of small ruminants in harsh environments. Therefore, in floodplain lands, it could increase the sustainability of livestock operations by providing high-quality forage during periods when herbaceous forage is limited or is of poor quality [28].

This work aims to evaluate the nutritive value and mineral element of the pod extracts of seven *Acacia* species (AMs—*A. mearnsii*, AL*—A. longifolia*, AMy*—A. melanoxylon*, AP*—A. pycnantha*, AD*—A. dealbata*, AR*—A. retinodes* and AC*—A. cyclops*) to be harnessed and used for different purposes, either in livestock farming as an alternative source of protein, or as minerals to be used in soil fertilization.

## 2. Results and Discussion

### 2.1. Nutritional Parameters

Forage plants provide animal feed sources all over the world. However, plant materials vary in the quantities of different nutritive components they deliver, so a careful analysis is necessary to optimize their use in animal food supplements. Forage plants can vary in the amounts of protein, fat, carbohydrate, fiber, and other macro or micronutrients present in the crude material. Additionally, herbivores vary in their need for these different nutritive components, influencing their dietary requirements, that change over time. Another important issue is the palatability of the forage plants and the structural compounds, such as lignin and fiber, that reduce the amount of plants ingested. In relation to the factors mentioned above, this research team thinks that knowledge of the nutritive value of *Acacia* pods could be used in ecosystem management strategies to increase the harvest of this invasive species.

On the other hand, maintaining soil health is also of crucial importance. Nutrient cycling and decomposition maintain soil biodiversity. Adding organic materials to the soil, such as crop residues or other plants, increases the organic matter content and provides nutrients for the crops and the soil organisms, stimulating microbial activity and, consequently, a better aggregation of the mineral particles.

Incorporating *Acacia* pods in animal feed or soil will be the focus of subsequent work, and the first step is a significant focus on their nutritional characteristics.

The average nutritional value of the pods of the different *Acacia* species studied is shown in Table 1, where it can be seen that the values for dry matter (DM) varied between 91.0 and 96.8% for AR and AD, respectively, and there were no significant differences between species. According to Sosa Rubio et al. [29], the DM variation depends on the region and season. The DM results of this work agree with the values obtained in other studies in which different species were used, namely 94.4% for *A. nilotica* [30] and 93.7% for *A. seyal* [18]. Concerning the amount of protein (% DM), the study with the pods showed statistically significant differences (*p* < 0.05), with protein ranging between 11.8 and 21.6%. AC and AMy presented the lowest concentration, while AR and AD had the highest contents. Finally, the higher values observed may be related to the age of the trees where the pods were harvested or to differences in nitrogen availability in the soil [31]. The amount of protein was similar to that reported by Abdalla et al. [32] (20.9%) and Zapata-Campos et al. [33] (17.2%) for *A. farnesiana*. The fat contents of the different *Acacia* pods varied between 0.8 and 1.8%, with the lowest values obtained in the AP specie and the highest in AL. Several authors have reported higher fat values in their studies with *A. nilotica*, ranging between 1.9 and 3.5% [18,25,33]. However, as far as we know, no studies evaluated the nutritional value of the pods studied in the present work. According to the results shown in Table 1, the fiber contents of the different species varied between 17.2 (AL) and 22.9% (AP). García-Winder et al. [25] and Uguru et al. [30] found lower values for fiber, 13.2 and 10.7%, respectively. However, Abdalla et al. [18] obtained *A. nilotica* values between 20.2 and 30.6%, which are more similar to those observed for our species.

Since the constituents of the plant cell wall are mainly cellulose, hemicelluloses, pectin, and lignin, and given the possibility of the pods serving as animal feed, we have also determined, in addition to fiber, the neutral detergent fiber (NDF), acid detergent fiber (ADF) and acid detergent lignin (ADL). The proportion of each is variable, depending on the species and growth stage of the plant [34].

NDF is composed mainly of cellulose, hemicelluloses and lignin, and can be considered a measure of plant cell wall material [35]. The acid detergent lignin (ADL) determination aimed at extracting all the cellulose from the samples to leave only indigestible constituents. The NDF contents ranged from 39.4% to 51.1% for the AL and AD species. According to the results, the average NDF of the different species was relatively higher than the NDF contents reported by Zapata-Campos et al. [33] (24.6%). However, these results are similar to and within the values reported by Hadi et al. [31] (43.0–54.5%). The amounts of ADF in pods varied between 25.7 (AD) and 35.2% (AM). The acid detergent lignin (ADL) determination aimed to extract all the cellulose from the samples, leaving only indigestible constituents. The same authors reported lower values in their studies, ranging from 21.8% to 29.8% [31] and 17.1% [33]. The highest ADL content was recorded for AMy (18.8%) and the lowest for AMs (7.2%) (Table 1). Our results are similar to those obtained by Garcia-Montes De Oca et al. [28] (8.5%).

The ash contents were obtained after the complete incineration of the samples in a muffle. Significant differences (*p* < 0.05) were observed in ash contents between species, and varied between 2.2 and 5.9%. Some authors reported that the ash contents could var between 3.5% [25,33], 3.9 to 5.4% [31] and 5.5 to 9.3%. In our study, AD presented the lowest ash contents, while AR presented the highest value.

### 2.2. Elemental Analysis

The mineral composition of plants varies according to the age of the plant, soil characteristics, plant species and varieties and climatic or seasonal conditions [36].

Table 2 and Table 3 show the mineral composition of the different *Acacia* pod species. The most abundant minerals in the samples were potassium (K), calcium (Ca) and magnesium (Mg), while cadmium (Cd) was the least abundant. Chromium (Cr) was also studied in the samples but was not detected in any analyzed samples. The NT content obtained using the Kjeldahl method varied among the species, with significant differences. NT values ranged from 1.8% to 3.4% for AC and AR, respectively. P had the lowest concentrations of the all macronutrients. All plant species showed a P content ranging from 0.0006 to 0.014 g/kg (Table 2), meaning the pods have low P content. Higher values were reported by Abdalla et al. [18] (2.9 g/kg). These differences may be related to the environmental conditions in which they were grown. The Ca results showed significant differences (*p* < 0.05). The values for Ca were similar in all pods except for AR and AMs. The values ranged from 2.8 g/kg to 10.07 g/kg for AMy and AR, respectively.

Comparing the global range of Ca content (7.6 to 13.6 g/kg) reported by Abdalla et al. [32] and Zapata-Campos et al. [33], it was found that all the species except for the AR showed lower values. However, the reported values [32,33] refer to a different *Acacia* species, which may justify the differences.

Furthermore, K ranged from 13.2 g/kg to 23.2 g/kg in the species (Table 2). These results are higher than those reported by Abdalla et al. [32] (11.7 to 13.4 g/kg), except for AC (13.15 g/kg). Zapata-Campos et al. [33] reported lower values (4.8 g/kg). In relation to the sodium (Na) content, significant differences were found among the studied species. The values ranged from 0.59 to 6.97 g/kg for AD and AL, respectively. Two species, AP and AL, had high concentrations of Na. Except for AL and AP, all the others had lower values than those reported by Zapata-Campos et al. [33] (1.2 g/kg). Significant differences were also observed in the Mg values. The species AD presented the lowest values (1.04 g/kg) and AR presented the highest (2.53 g/kg). Abdalla et al. [32] reported higher Mg values than ours (3.5 g/kg). However, Zapata-Campos et al. [33] reported lower values (1.1 g/kg).

The AR showed the highest concentrations of NT, P, Ca, K and Mg; only Na had lower values than the other samples.

Looking at Table 3, we can observe that iron (Fe) concentrations were similar in all pods except those of AMy. The values ranged from 25.7 to 46.8 mg/kg. These results are similar to those reported by Zapata-Campos et al. [33] (40.3 mg/kg). The seven green pod species differ in manganese (Mn) composition, and therefore significant differences were observed (*p* < 0.05). AP and AMy showed the highest concentration (50.2 mg/kg and 48.7 mg/kg), respectively, while the pods of AC showed the lowest concentration of Mn. The copper (Cu) values are similar between the species. AMy and AC were the species that presented higher values (14.6 mg/kg and 13.5 mg/kg). These values agree with those reported by Zapata-Campos et al. [33] (8.9 mg/kg). Abdalla et al. [32] obtained relatively low values for Cu (6.9 mg/kg). In relation to the zinc (Zn) contents, significant differences were found among the species; AR and AL (23.13 and 20.24 mg/kg) showed the highest concentrations of Zn, while AC showed the lowest results (15.84 mg/kg). The amounts of lead (Pb) in pods varied between 1.9 mg/kg (AR) and 4.83 (AP). After Cd, Pb was the mineral that showed the lowest concentrations. Regarding nickel (Ni) values, AD had the highest concentration (8.0 mg/kg), compared to AMs which had the lowest (4.5 mg/kg).

Concerning macro or micronutrients, Pb, Cd and Cr are always phytotoxic, and are found in very small amounts. The amounts of Pb in pods ranged between 1.9 mg/kg (AR) and 4.83 (AP). As aforementioned, Cd effectively showed low values for the different species, ranging from 0.04 mg/kg to 0.2 mg/kg for AP and AMy. Since Pb, Cd and Cr were found in the pods in such small amounts, these toxic mineral elements were not expected to negatively affect animal health or contribute to soil contamination.

**Figure 1:**
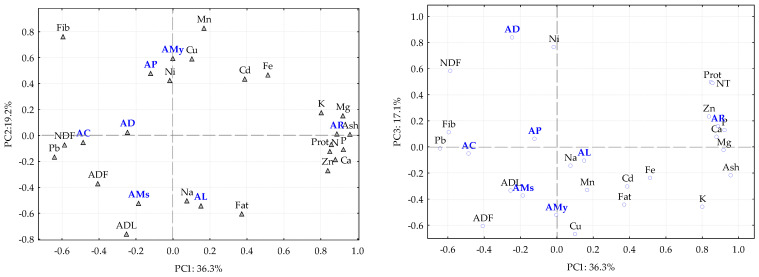
Scores and loadings of the principal component analysis (PC1 vs. PC2 and PC1 vs. PC3) for different *Acacia* species and the analytical parameters measured. AMy—*A. melanoxylon*, AD—*A. dealbata*, AC—*A. cyclops*, AR—*A. retinodes*, AMs—*A. mearnsii*, AP—*A. pycnantha*, AL—*A. longifolia*, Pro—protein, Fat—fat, Fib—fiber, NDF—neutral detergent fiber, ADF—acid detergent fiber, ADL—acid detergent lignin, Ca—calcium, K—potassium, Na—sodium, Mg—magnesium, Fe—iron, Mn—manganese, Cu—copper, Zn—zinc, Pb—lead, Ni—nickel, Cd—cadmium, NT—total nitrogen, P—phosphorus.

To complement the previous results, a principal component analysis (PCA) was performed with the nutritional and mineral content of each green pod (Figure 1) in which the three first components explain 72.6% of the total variation (PC1= 36.3%; PC2 = 19.2%; PC3 = 17.1%).

The first component, which explains 36.3% of the total variation, clearly separates the green pods of AR from the other species. This differentiation is related to a higher amount of the minerals K, Mg, P, NT, Ca and Zn and a higher amount of ash (associated with the amount of mineral content) and protein. For component 1, AC, which has a higher amount of NDF and Pb, appears opposite AR. Component 2, which explains 19.2% of the total variation, is responsible for the discrimination between two groups of *Acacia* pods, the first one comprising AP and AMy and the second one AMs and AL. AP and AMy are characterized by a higher amount of Cu, Mn and fiber contents, and the second group presents a higher amount of Na, ADL, fat and ADF. AD is discriminated by component 3 (17.1% of the total variation) given the amount of Ni.

Table 4 represents the decision tree made, first with the nutritional parameters and then with the mineral content, using the Gini index. Decision trees are machine learning algorithms that implement the splitting conditions at each node and break down the training data into subcategories of output variables of the same class [37]. In this case, many assay replicates are needed if many variables exist in the models. The number of replicates in this study is four, and therefore two tests were made separately.

**Figure 2:**
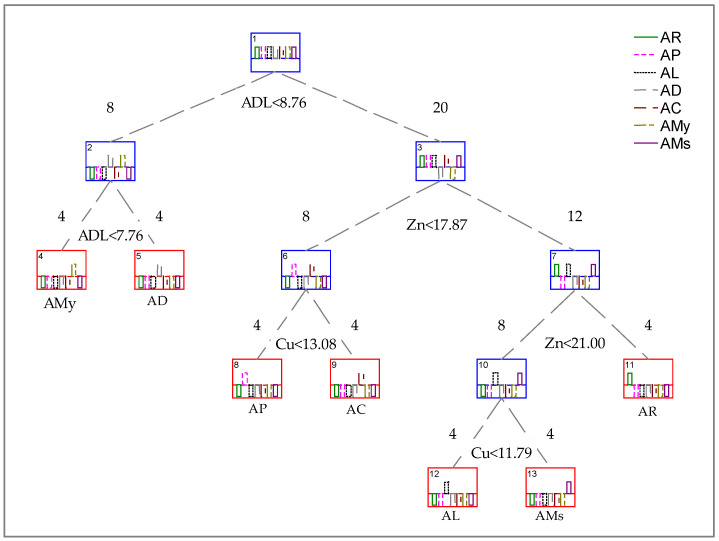
Decision tree results for the discriminant parameters of *Acacia* green pods with the nutritional and mineral parameters with the strongest influence. Decision nodes are represented in blue and terminal nodes are in red. AMy—*A. melanoxylon*; AD—*A. dealbata*; AC—*A. cyclops*; AR—*A. retinodes*; AMs—*A. mearnsii*; AP—*A. pycnantha*; AL—*A. longifolia*, ADL—acid detergent lignin, Zn—zinc, Cu—copper.

The two groups analyzed lead us to the outcome of the more influenced variables that will be used to construct the final decision tree (Figure 2).

In Table 4, it is possible to observe that all nodes separate the four samples of each group with reasonable accuracy. Regarding the nutritional value, the split variables are fat, Fib, ADL and Ash. Concerning the mineral contents, the split variables are Cu, K and Zn.

Figure 2 represents the decision tree made with the selected parameters in Table 4. With this analysis, it is possible to identify four distinct groups. AR can be distinguished from the others by its amount of Zn. Then, with an amount of Zn lower than 21 mg/Kg, there is a group composed of AL and AMs *Acacia* green pods (separated by the Cu concentration) and with an amount of Zn lower than 17.8 mg/Kg, there is the group composed of AP and AC (also divided by Cu concentration). Conversely, the amount of ADL discriminates the above groups from those composed of AMy and AD. Their concentration in ADL could also distinguish AMy and AD.

It can be concluded that, concerning the analyzed parameters, ADL, Zn and Cu contents are the factors with a more significant influence in discriminating the green pod species studied.

### 2.3. Spectroscopic Analysis

**Figure 3:**
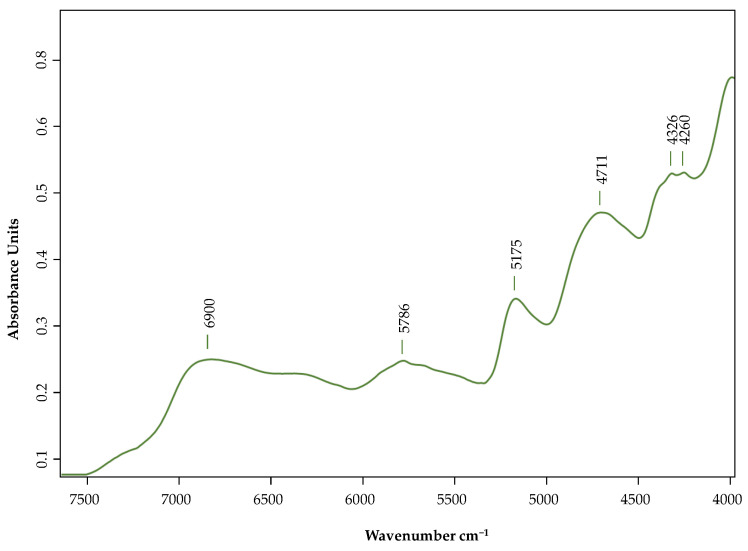
NIR spectra of *Acacia* green pods obtained from freeze-dried powder.

Near-infrared spectroscopy (NIR) spectra are shown in Figure 3 and present representative bands in the 7500–3700 cm^−1^.

The peaks observed in our samples were mainly due to organic the molecules’ primary structural components due to the stretch or deformation vibration of N-H, C-H, O-H and C=O bonds [38,39,40]. The large band at 6900 cm^−1^ can be assigned to the first overtone of N-H and O-H stretching vibrations of protein and carbohydrates [39,41]. At around 5786 cm^−1^ the first overtone of C-H stretching vibrations is observed [42]. At 5175 cm^−1^, the peak was assigned to C–O stretching as the second overtone of carbohydrates and to C–H stretching as the first overtone of cellulose, lignin and other carbohydrates of plant fiber and C=O functionality [38,43]. The peaks at 4711 cm^−1^ and 4326 cm^−1^ are assigned to the N–H symmetric stretching of amide II of proteins and the O-H bond combined with the C-O bond, the C-H bond, and the C-H bond combined with the C-H bond, respectively, are sensitive to phenolic compounds and proteins [39,42]. The band at 4260 cm^−1^ is the combination band of C–H stretching vibrations [44].

**Figure 4:**
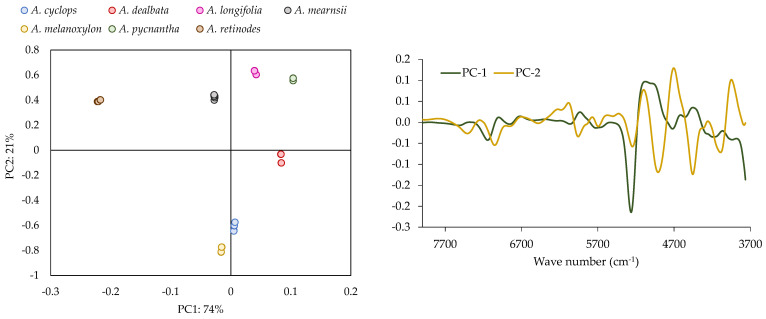
Principal Component Analysis performed with NIR (Scores—(**left side**) and Loading—(**right side**)) performed using first derivative Savitzky—Golay spectra transforms with 17 smoothing points.

PCA has been used to discriminate the differences between different species of *Acacia* pods in terms of qualitative analysis (Figure 4). This PCA (mean-centered) is performed with the spectral information acquired with the NIR, using the algorithm derived from the Savitzky—Golay filter with 17 smoothing points.

Concerning the PCA results, a clear separation between species is observed. These results showed that NIR spectroscopy is a promising technique for discriminating between species, and in Figure 4 (right), the more relevant regions for this discrimination are identified.

## 3. Materials and Methods

### 3.1. Plant and Soil

In May and April of 2021, *Acacia* pods of various species (AD, AC, AMs, AL, AR, AMy and AP) were collected in different regions of Portugal (Table 5). The pods were collected from two individuals for each species. Different regions were used because the *Acacia* species have heterogeneous distribution in Portuguese territory and grow in different areas and soils in the country. As the first step, areas where each specie was more plentiful were selected and as the second step, areas where we had permits for collecting and which were close to each other were selected.

The World Reference Base (WRB) soil classification System was used for the soil classification used in this work with a scale of 1:5,000,000 [45,46].

**Figure 5:**
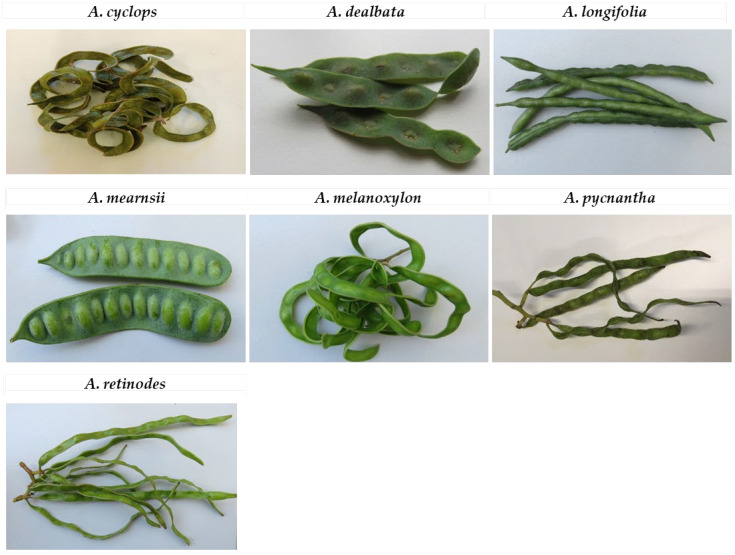
Different Acacia green pod samples studied collected at the same maturity stage.

In Figure 5, we can see the selected pods after they have been harvested.

### 3.2. Sample Preparation

After collecting the pods, they were frozen (Binder) at −80 °C and two different methods were used according to the determination.

For nutritional parameters, pod samples were dried in a forced draught oven (Memmert UL 60, Memmert GmbH, Schwabach, Germany) at 65 °C (±5 °C) for 24 h to determine moisture. Samples were milled through a laboratory mill with a 1 mm sieve and stored in tightly sealed plastic bottles for later chemical analysis. The powder obtained was used for all nutritional determinations performed, other than that for moisture when the samples were placed on a tray as collected to aid dehydration.

For the elemental analysis, a freeze-drier (Mitsubishi Electric Got2000, Tokyo, Japan) was used for 48 h at a pressure of 0.180 mbar to remove all water in the samples.

In both methods, after drying, the samples were reduced to powder (<2 mm) using a laboratory cutting mill (Polymix, PX-MFC 90 D, Malters, Switzerland).

### 3.3. Nutritional Parameters

The nutritional composition analysis of Acacia was performed according to the AOAC Official Methods of Analysis [47] in triplicate.

For total dry matter (DM), they were dried at 103 ± 2 °C for 5–6 h in a forced draught oven (Memmert UL 60, Memmert GmbH, Schwabach, Germany)and total ash content (Ash) was determined by incineration in a muffle furnace (Nabertherm L15 C 6, Nabertherm GmbH, Lilienthal, Germany) for 6–7 h at 550 ± 50 °C. Fat was determined by the Soxtec method (Soxtec System HT 1043 Extraction Unit (Tecator, Hoganas, Sweden) and heating unit Soxtec System HT 1046 Service Unit) using petroleum ether as a solvent. The Kjeldahl method allowed us to obtain the total nitrogen in the samples, and protein was calculated by using the nitrogen percentage and a conversion factor of 6.25. In the Kjeldahl method for protein, the fresh sample was firstly digested (Digestion System 6 (1007)—Tecator, Hoganas, Sweden) and after the mixture was distilled using the Kjeltec System 1026 Distilling Unit—Tecator (Tecator, Hoganas, Sweden). The Weende method determined the fibre using the Fibertec System M, 1020 Hot Extractor (Tecator, Hoganas, Sweden). NDF, ADF and ADL were performed using the procedure described in [35]. To determine the NDF, the samples were hydrolyzed with a neutral detergent solution (sodium lauryl sulfate-based). The purpose was to extract all constituents that were not part of the fiber from the samples, leaving the constituents of the plant cell wall.

The ADF was determined after hydrolyzing the samples with an acid detergent solution (cetyltrimethylammonium bromide-based). The main objective was removing all constituents that were not part of the fiber or the hemicellulose from the samples.

The ADF residue was subjected to a 72% sulfuric acid solution to determine ADL.

### 3.4. Elemental Analysis

The extraction methodology used to determine macro and micronutrients in samples of plant material enables the determination of several total mineral elements, namely, Ca, Mg, P, K, Mn, Cu, Pb, Cd, Zn, Fe, Cr, Ni and Na. These elements are quantified by atomic absorption spectrophotometry (Thermo Scientific Series iCE 3000, Waltham, Göteborg, Sweden) except for the element P, which must be quantified by molecular absorption spectrophotometry (spectrophotometer Thermo Electron Corporation evolution 300 LC, Waltham, MA, USA). This methodology is based on extracting these elements in samples after incineration using digestion with hydrochloric acid.

### 3.5. Extraction Conditions

Samples (10 g powder) of the seven A. species were extracted with 100 mL of 99% ethanol on an orbital plate shaker (VWR, Advanced Digital Shaker, Germany) for 24 h with constant stirring. After filtration, the extracts were centrifuged in (VWR, Mega Star 600R, Germany) (4000 g-forces, 20 min), and the supernatant was removed. All extractions were duplicated, and all subsequent measurements and analyses were performed in triplicate.

### 3.6. Spectroscopic Analysis

Spectra were acquired using a NIR spectrometer (MPA Bruker) in a transmitted light mode with 1 mm quartz cells. Four spectra per sample were obtained with sixty-four scans per spectrum, with a spectral resolution of 16 cm^−1^ in spectral region of 7500 to 3700 cm^−1^. The background was performed between samples.

### 3.7. Statistical Analysis

A one-way variance analysis (ANOVA) was performed to estimate the differences in the different parameters analyzed in crude material using the different species as a factor. The Schefee test was applied, with a t-critical value of α = 0.05, to determine whether the individual means of each parameter differed. Classification trees were used to understand which variables are more representative in differentiating Acacia species. At each classification tree step, the most informative parameters were selected as the source of the (sub)tree. The current training set was split into subsets according to the values of the selected attribute. The selected parameter was considered a good discriminator if the branches separated all the measurements observed for each sample group.

Principal component analysis was performed with NIR spectral data collected in which different mathematical pre-processes were tested, namely, Savitzky–Golay first and second derivative, standard normal variate (SNV) transformation, multiplicative scatter correction (MSC) and different combinations of these treatments.

Software STATISTICA 7 (StatSoft. Inc. Tulsa, OK, USA) was used to perform PCA with analytical data. For spectral data analysis UnscramblerX 10.5 (CAMO, Oslo, Norway) and OPUS^®^, version: 7.5.18 (Bruker Optik, Rosenheim, Germany) were used.

## 4. Conclusions

This work evaluates the nutritive value and mineral element content of the pod extracts of seven Acacia species to be harnessed and used for different purposes, either in livestock farming, as an alternative source of protein, or as a mineral source in soil fertilization. The study showed that the pods of Acacia could be used as mineral corrections. The most abundant minerals in the samples were K, Ca and Mg, while Cd was the least abundant. A. retinodes is a good source of N (3.4%), P (0.014 g/kg), Ca (10.07 g/kg), K (23.19 g/kg) and Mg (2.53 g/kg). The amount of protein in the pods ranged between 11.8 (for AC) and 21.6% (for AR), the fat content between 0.8 (for AP) and 1.8% (for AL) and the acid detergent fiber between 25.7 (AD) and 35.1% (AMy). NIR spectroscopy appeared to be a promising technique for discriminating between species. Taking into account the results presented, it is can be affirmed that it is possible to use the pods as a source of protein and other nutrients for animals. Therefore, this could be a new method of using this waste from management actions taken to control the Acacia species and could also promote their harvest in order to help in the control of these invasive species. However, studies evaluating bioaccessibility, bioavailability and toxicity are necessary and will be the aim of future research. We also want to highlight that this is the first study of its kind that evaluates the nutritional profile of pods from different species of Acacia.

## Figures and Tables

**Table 1:** Nutritional value of *Acacia* pod species, on a dry matter basis (% in DM) (mean ± standard deviation).

Species	DM (% in DM)	Prot (% in DM)	Fat (% in DM)	Fib (% in DM)	NDF (% in DM)	ADF (% in DM)	ADL (% in DM)	Ash (% in DM)
AMy	91.41 ± 0.02	13.68 ± 0.22 ^b^	1.48 ± 0.01 ^c^	19.51 ± 0.15 ^bc^	44.41 ± 0.15 ^c^	35.17 ± 0.37 ^c^	18.82 ± 0.91 ^e^	3.24 ± 0.08 ^c^
AD	96.79 ± 0.09	17.68 ± 0.35 ^d^	1.09 ± 0.00 ^b^	22.11 ± 0.29 ^d^	51.17 ± 0.67 ^d^	25.74 ± 0.18 ^a^	8.28 ± 0.59 ^a^	2.25 ± 0.01 ^a^
AC	96.22 ± 0.09	11.82 ± 0.06 ^a^	1.10 ± 0.02 ^b^	21.16 ± 0.12 ^c^	50.15 ± 0.05 ^d^	32.88 ± 1.01 ^c^	15.88 ± 0.44 ^d^	2.62 ± 0.03 ^b^
AR	91.03 ± 0.10	21.66 ± 0.27 ^e^	1.37 ± 0.00 ^c^	17.87 ± 0.28 ^ab^	41.72 ± 0.41 ^ab^	26.92 ± 0.83 ^a^	10.82 ± 0.39 ^bc^	5.89 ± 0.02 ^e^
AMs	96.20 ± 0.02	14.11 ± 0.17 ^bc^	1.42 ± 0.05 ^c^	22.59 ± 0.04 ^d^	40.85 ± 0.43 ^ab^	29.78 ± 0.35 ^b^	7.25 ± 0.58 ^a^	3.69 ± 0.00 ^d^
AP	93.21 ± 0.02	14.96 ± 0.16 ^c^	0.87 ± 0.00 ^a^	22.87 ± 0.16 ^d^	41.67 ± 0.08 ^ab^	25.83 ± 0.40 ^a^	8.87 ± 0.04 ^ab^	3.33 ± 0.02 ^c^
AL	93.80 ± 0.08	16.95 ± 0.08 ^d^	1.82 ± 0.10 ^d^	17.16 ± 0.91 ^a^	39.44 ± 0.25 ^a^	27.24 ± 0.40 ^ab^	12.69 ± 0.22 ^c^	3.84 ± 0.05 ^d^

AMy—*A. melanoxylon*, AD—*A. dealbata*, AC—*A. cyclops*, AR—*A. retinodes*, AMs—*A. mearnsii*, AP—*A. pycnantha*, AL—*A. longifolia*. Prot—protein, Fat—fat, Fib—fiber, NDF—neutral detergent fiber, ADF—acid detergent fiber, ADL—acid detergent lignin, DM—Dry Matter. Different letters in the same column denote significant differences between *Acacia* pods by the Scheffe test (*p* < 0.05) for each analytical determination.

**Table 2:** The concentration of total nitrogen (NT); phosphorus (P); calcium (Ca); potassium (K); sodium (Na), and magnesium (Mg) for *Acacia* species.

Species	NT (%)	P (mg/kg)	Ca (mg/kg)	K (mg/kg)	Na (mg/kg)	Mg (mg/kg)
AMy	2.18 ± 0.03 ^b^	0.09 ± 0.00 ^bc^	2803.61 ± 87.31 ^a^	18,845.42 ± 398.00 ^e^	748.86 ± 17.19 ^ab^	1454.36 ± 67.37 ^c^
AD	2.83 ± 0.06 ^e^	0.09 ± 0.00 ^bc^	3082.52 ± 50.33 ^a^	10,957.26 ± 1528.57 ^a^	593.86 ± 50.90 ^a^	1038.80 ± 36.61 ^a^
AC	1.89 ± 0.01 ^a^	0.06 ± 0.00 ^a^	3240.21 ± 82.72 ^ab^	13,154.26 ± 602.65 ^b^	1415.35 ± 35.22 ^c^	1288.24 ± 1.99 ^b^
AR	3.46 ± 0.04 ^f^	0.14 ± 0.00 ^e^	10,069.31 ± 582.88 ^e^	23,196.54 ± 1132.75 ^f^	715.44 ± 34.33 ^ab^	2532.32 ± 102.94 ^d^
AMs	2.26 ± 0.03 ^b^	0.10 ± 0.00 ^d^	4833.96 ± 33.99 ^d^	17,569.05 ± 422.92 ^de^	986.52 ± 40.38 ^b^	1061.40 ± 27.99 ^a^
AP	2.40 ± 0.03 ^c^	0.09 ± 0.00 ^b^	3656.00 ± 181.65 ^bc^	16,231.81 ± 555.47 ^cd^	1756.47 ± 56.71 ^c^	1500.28 ± 50.29 ^c^
AL	2.71 ± 0.01 ^d^	0.09 ± 0.00 ^c^	4064.88 ± 61.01 ^c^	14,673.65 ± 180.52 ^bc^	6975.65 ± 385.21 ^d^	1531.81 ± 26.91 ^c^

AMy—*A. melanoxylon*, AD—*A. dealbata*, AC—*A. cyclops*, AR—*A. retinodes*, AMs—*A. mearnsii*, AP—*A. pycnantha*, AL—*A. longifolia*. Different letters in the same column denote significant differences between *Acacia* pods by the Scheffe test (*p* < 0.05) for each analytical determination.

**Table 3:** The concentration of iron (Fe); manganese (Mn); copper (Cu); zinc (Zn); lead (Pb); nickel (Ni), and cadmium (Cd) for *Acacia* species.

(mg/Kg)	AMy	AD	AC	AR	AMs	AP	AL
Fe	45.54 ± 15.13 ^c^	30.61 ± 1.68 ^ab^	30.35 ± 1.99 ^ab^	44.13 ± 4.35 ^bc^	25.70 ± 0.13 ^a^	46.50 ± 0.72 ^c^	46.85 ± 0.88 ^c^
Mn	48.78 ± 2.55 ^d^	15.04 ± 0.68 ^a^	13.15 ± 0.34 ^a^	25.93 ± 1.83 ^c^	14.73 ± 0.31 ^a^	50.20 ± 0.91 ^d^	19.15 ± 0.21 ^b^
Cu	14.62 ± 0.86 ^e^	10.75 ± 0.10 ^a^	13.49 ± 0.22 ^d^	12.99 ± 0.72 ^cd^	12.13 ± 0.27 ^bc^	12.73 ± 0.14 ^cd^	11.55 ± 0.12 ^ab^
Zn	17.50 ± 2.42 ^ab^	19.30 ± 0.32 ^b^	15.84 ± 0.31 ^a^	23.13 ± 2.60 ^c^	18.67 ± 0.54 ^ab^	17.00 ± 0.64 ^ab^	20.24 ± 0.60 ^bc^
Pb	3.80 ± 0.12 ^ab^	4.63 ± 1.05 ^b^	4.12 ± 0.18 ^ab^	1.94 ± 2.30 ^a^	4.93 ± 0.55 ^b^	4.83 ± 0.85 ^b^	4.70 ± 0.12 ^b^
Ni	5.95 ± 0.10 ^bc^	8.03 ± 0.66 ^e^	7.20 ± 0.08 ^de^	7.02 ± 1.02 ^cde^	4.56 ± 0.12 ^a^	6.73 ± 0.07 ^bcd^	5.52 ± 0.62 ^ab^
Cd	0.25 ± 0.03 ^c^	0.10 ± 0.02 ^ab^	0.08 ± 0.01 ^ab^	0.19 ± 0.08 ^bc^	0.11 ± 0.05 ^ab^	0.04 ± 0.04 ^a^	0.04 ± 0.04 ^a^

AMy—*A. melanoxylon*, AD—*A. dealbata*, AC—*A. cyclops*, AR—*A. retinodes*, AMs—*A. mearnsii*, AP—*A. pycnantha*, AL—*A. longifolia*. Different letters in the same line denote significant differences between *Acacia* pods by the Scheffe test (*p* < 0.05) for each analytical determination.

**Table 4:** Tree structure for *Acacia* green pods, nutritional and elemental content separately, child nodes, n in observed class, predicted class and split condition for each node.

	Node	Left Branch	Right Branch	n in Class							Predicted Class	Split Constant	Split Variable
				AR	AP	AL	AD	AC	AMy	AMs			
Nutritional content	1	2	3	4	4	4	4	4	4	4	AR	−1.0875	Fat
	2	4	5	0	4	0	4	0	0	0	AP	−22.5317	Fib
	3	6	7	4	0	4	0	4	4	4	AR	−11.8166	ADL
	4			0	0	0	4	0	0	0	AD		
	5			0	4	0	0	0	0	0	AP		
	6	8	9	4	0	0	0	0	4	0	AR	−1.3780	Fat
	7	10	11	0	0	4	0	4	0	4	AC	−3.5536	Ash
	8			4	0	0	0	0	0	0	AR		
	9			0	0	0	0	0	4	0	AMy		
	10	12	13	0	0	0	0	4	0	4	AC	−1.2900	Fat
	11			0	0	4	0	0	0	0	AL		
	12			0	0	0	0	4	0	0	AC		
	13			0	0	0	0	0	0	4	AMs		
Mineral content	1	2	3	4	4	4	4	4	4	4	AR	−11.8	Cu
	2	4	5	0	0	4	4	0	0	0	AD	−13,292.1	K
	3	6	7	4	4	0	0	4	4	4	AR	−16,947.3	K
	4			0	0	0	4	0	0	0	AD		
	5			0	0	4	0	0	0	0	AL		
	6	8	9	0	4	0	0	4	0	0	AP	−14,709.7	K
	7	10	11	4	0	0	0	0	4	4	AR	−20.2	Zn
	8			0	0	0	0	4	0	0	AC		
	9			0	4	0	0	0	0	0	AP		
	10	12	13	0	0	0	0	0	4	4	AMy	−18,216.0	K
	11			4	0	0	0	0	0	0	AR		
	12			0	0	0	0	0	0	4	AMs		
	13			0	0	0	0	0	4	0	AMy		

AMy—*A. melanoxylon,* AD—*A. dealbata,* AC—*A. cyclops,* AR—*A. retinodes,* AMs—*A. mearnsii,* AP—*A. pycnantha,* AL—*A. longifolia*, Fat—fat, Fib—fiber, ADL—acid detergent lignin, Cu—copper, K—potassium, Zn—zinc.

**Table 5:** Origin of *Acacia* species.

Specie	Origin	Collect Date	Longitude	Latitude	Altitude (m)	Soil Classification
AMy	Vimeiro, Alcobaça	08/05/2021	39.4904	−9.0367	163	Cambisol
AD	Praia de Paredes da Vitoria, Alcobaça	03/04/2021	39.7025	−9.0483	24	Podzois
AC	Tapada da Ajuda, Lisboa	01/04/2021	38.7121	−9.1908	86	Vertisol
AR	Lagoa, Pinhal de Leiria	01/04/2021	39.7478	−8.9533	57	Podzois
AMs	Vimeiro, Alcobaça	08/05/2021	39.4904	−9.0367	163	Cambisol
AP	Silvares, Fundão	08/05/2021	40.1507	−7.6444	477	Regossolos
AL	Tercena, Queluz	08/05/2021	38.7425	−9.2822	90	Vertisol

AMy—*A. melanoxylon*, AD–*A. dealbata*, AC—*A. cyclops*, AR—*A. retinodes*, AMs—*A. mearnsii*, AP—*A. pycnantha*, AL—*A. longifolia*.

## Data Availability

Not applicable.
